# Alternative Forages as Roughage for Ruminant: Nutritional Characteristics and Digestibility of Six Exotic Plants in Azores Archipelago

**DOI:** 10.3390/ani12243587

**Published:** 2022-12-19

**Authors:** Helder P. B. Nunes, Sofia Teixeira, Cristiana S. A. M. Maduro Dias, Alfredo E. S. Borba

**Affiliations:** Institute of Agricultural and Environmental Research and Technology (IITAA), Faculty of Agricultural and Environmental Sciences, University of the Azores, 9700-042 Angra do Heroísmo, Portugal

**Keywords:** alternative forages, valorization forages, livestock, sustainability feed, animal nutrition, climate change

## Abstract

**Simple Summary:**

The potential use of exotic plants in animal feed provides a source of fiber for grazing ruminants when there is a shortage of pasture. The objective of this research was to evaluate the nutritional value, digestibility, and gas production potential of six exotic plants present in the Azores. Samples of these plants were collected and their chemical composition and potential as forage food were evaluated. Data analysis included univariate and multivariate methods. The results obtained showed variations among the studied forages in terms of crude protein, and fibers: NDF, ADF, and ADL. The gross, metabolizable, and digestible energy of *E. globulus* and *C. japonica* showed higher values than the other plants. *P. undulatum* with a relative food value of 92.12% stood out from the other plants. In gas production, the values of gas produced by *A. donax* stand out, as the species that presented the highest gas production, observing a significant difference) for the other plants. *P. undulatum* stands out for presenting good quality in the RFV index and *A. donax* for having good digestibility, both can be used as forage in periods of greater scarcity of pastures.

**Abstract:**

This study aimed to evaluate the nutritional potential of unconventional plants: *Pittosporum undulatum*, *Cryptomeria japonica*, *Acacia melanoxylon*, *Hedychium gardnerianum*, *Eucalyptus globulus*, and *Arundo donax*, as an alternative roughage for ruminants. Chemical composition, gross energy, in vitro gas production, kinetics, and digestibility of dry matter and organic matter in vitro were determined for each species. The obtained results showed variations between the studied forages concerning crude protein, and the different fiber fractions: NDF, ADF, and ADL The *P. undulatum* with a relative food value of 92.12%, showed a significant difference compared to the other species under study. After 96 h of incubation, the plants that produced, on average, less in vitro gas were *A. melanoxylon* and *E. globulus*. Among the studied species, *A. donax* stands out as the species that presented the highest gas production, with 31.53 mL. 200 mg^−1^ DM, observing a significant difference compared to the other plants. This is a reflection of it having the highest DMD (60.44 ± 1.22%) as well. *P. undulatum* was the species with the longest colonization time (4.8 h). Among the plants studied, we highlight *P. undulatum* as presenting a good quality in the RFV index and *A. donax* as having good digestibility. Both can be used as roughage in periods of greater shortage of pastures.

## 1. Introduction

The forecasts of several climate change models for the Azores archipelago indicate an increase in average air temperature and a decrease in average annual precipitation, also foreseeing irregularity in the distribution of precipitation, i.e., the existence of periods of heavy rainfall, alternating with long periods of drought [1]. Regions where animal production systems are based on pasture, as is the case of the Azores, are highly dependent on the climate for their production, and are very vulnerable to climate change, with these regions being the ones that suffer most from drought. As a result, the demand for unconventional animal feed sources has been growing worldwide [2,3]. Given the current economic scenario and the scarcity of food, which has displayed a tendency to worsen, the importation of fiber to the outermost regions becomes unaffordable for most producers. Furthermore, from a resource optimization perspective, it is important to value the endogenous resources of each region, especially those that do not compete with human food [4]. In this context, the valorization of unconventional forages gains preponderance, given the importance of shrub and tree foliage in the feeding of ruminants in extensive systems [3]. The use of unconventional forages has been the subject of research, namely the study of the forages’ chemical composition, nutritional value, and digestibility to enhance these resources in the nutrition of ruminants. The valorization of these resources is an opportunity to combat desertification in arid areas and to control invasive plants in native forests [5], as it is possible to use some of these plants as roughage foods since they are rich in fiber and generally present higher levels of protein and minerals than the straw supplied to ruminants [6].The major handicap for the use of these unconventional forages is the existence of some groups of secondary metabolites in the plants’ chemical composition, of which the terpenoids, the flavonoids, the phenolic, and alkaloid compounds stand out. These can interfere with digestibility [7]. In high concentrations, some secondary metabolites can produce toxic effects in different systems, such as endocrine, immunological, and reproductive [8]. However, the use of unconventional forages in ruminants’ feed can bring benefits to the animal because they have secondary metabolites capable of binding proteins in the rumen, making it escape ruminal degradation, and these metabolites can only be digested in the intestine, increasing the amount of protein absorbed by the animal [6]. In addition, secondary metabolites can alter the balance of microorganisms in the rumen which will have a direct influence on the fermentative potential of the fiber present in the food [9]. Thus, the use of unconventional plants in the feeding of ruminants can contribute to a natural reduction in the production and enteric emission of greenhouse gases produced by ruminant production systems; this change will, namely, reduce methane and lead to an increase in rumen fermentation, through the decrease in the use of metabolizable energy through the loss of gross energy, thereby increasing animal production [10].

However, it is first necessary to strengthen the knowledge of the nutritional properties of these exotic species, as well as their capacity for digestibility and how they can adapt to the regional model of livestock production. The objective of this study was to evaluate the nutritional characteristics of six unconventional plants, *Pittosporum undulatum*, *Cryptomeria japonica*, *Acacia melanoxylon*, *Hedychium gardnerianum*, *Eucalyptus globulus*, and *Arundo donax* and their potential as nutritional alternatives for grazing ruminants.

## 2. Materials and Methods

The current study was conducted in the Animal Nutrition Laboratory, University of the Azores, Portugal.

### 2.1. Forage Collection and Preparation

The plant materials (leaves) of six different plants, *P. undulatum*, *C. japonica*, *A. melanoxylon*, *H. gardnerianum*, *E. globulus*, and *A. donax* were collected from the Terra Chã forest (38°41’ N 27°14’ W, at 319 m altitude) on Terceira Island, Azores, between November and February. All plants were harvested before flowering. Three different samples from each plant were harvested manually, consisting of the parts of the plant animals normally eat. Samples were chopped into small pieces, then dried at 65 °C in a forced air oven for 72 h, ground through a 1mm screen using a Retsch mill (GmbH, 5657 Hann, Germany), and stored in tightly closed bags till use.

### 2.2. Chemical Analysis

Chemical analysis was performed in triplicate. The dry matter (DM) contents in forage were determined by the Weende system (DM, method 930.15)), crude protein (CP, method 954.01), ether extract (EE, method 920.39), and total ash method (942.05) were determined according to the standard methods of AOAC [11]. The DM content of forage was determined by drying the samples at 68 °C until a steady weight and placing them in a forced air oven at 105 °C for 24 h. Crude protein (CP) was determined by a standard micro-Kjeldahl method using digestion equipment (Kjeldatherm System KT 40, Gerhart Laboratory Instruments, Bonn, Germany) and an automated Kjeltec 2300 Auto-analyzer apparatus for distillation and titration (Foss Electric, Copenhagen, Denmark.) The CP was calculated by multiplying the total nitrogen by 6.25. The ether extract was measured by refluxing forage samples with petroleum ether in a Soxhlet system (Büchi B-810, Flawil, Switzerland). The total ash was evaluated by igniting samples in a muffle furnace at 500 °C for 12 h. Neutral detergent fiber (NDF), acid detergent fiber (ADF), and acid detergent lignin (ADL) were determined according to the method used by Goering and Van Soest [12]. In vitro digestibility was determined according to the method of Tilley and Terry [13], modified by Alexander and McGowan [14]. The rumen liquid was obtained as described by Borba et al. [15], from a local slaughterhouse bovine, where the following conditions were observed: For each experiment, rumen were collected from 5 healthy dairy cattle (Holstein-Friesian); the animals had been fed ryegrass. Rumen fluid was collected within 10 min of slaughter, filtered through cheesecloth, and preserved at 38 °C under anaerobic conditions, being delivered to the animal nutrition laboratory within 30 min after it was collected.

### 2.3. In vitro Gas Production

The in vitro gas production (IVGP) technique [16] was followed for ruminal fermentation determination in glass syringes (Model Fortuna from Germany); it was also used to evaluate the potential of feeds to produce greenhouse gas. Rumen liquor was collected as described by Borba et al. [15]. For each oven-dried sample, 200 mg was weighed, in triplicate, for 100 mL calibrated glass syringes fitted with plungers. Three blank syringes were incubated with only 30 mL of buffered rumen fluid. Blanks were used for each inoculum to measure the fraction of total gas production due to the substrate in the inoculum, and these values were subtracted from the total to obtain the net GP. All treatments for each essay were incubated simultaneously in all runs. The preparation of buffer solutions and the rumen inoculum was performed as described by Menke [17]. The initial gas volume was recorded after 4, 8, 12, 24, 48, 72, and 96 h of incubation. To study the kinetics of gas production used exponential equation (1) of Ørskov and McDonald [18], implemented in the NEWAY computer package, was used:

*p* = a + b (1 − exp^−ct^), (1)

where: *p* represents the gas production at time t, the values of a, b and c represent constant values in the exponential equation, a + b is the total potential gas production (mL g^−1^ DM), and c the rate constant. This equation was chosen because its constants and derivatives have been the object of constant study, and great results have been observed when applying this equation to various unconventional forages [19].

### 2.4. Gross Energy Determination

The gross energy (GE) was determined using an adiabatic calorimeter bomb (IKA-WERKE C5003, Staufen, Germany). All GE determinations were performed in triplicate. 

### 2.5. Relative Feed Value Determination

The RFV of leaves was calculated according to Rohweder [20] using the following equations (2):

RFV = (DMD × DMI)/1.29(2)

This index is useful to evaluate forage quality since it combines both feed digestibility (from %ADF) and the intake potential (from %NDF) [21]. In line with the Quality Grading Standard assigned by The Hay Marketing Task Force of the American Forage and Grassland Council, the RFV was assessed as roughages based on: prime >151; 1st (premium)—151−125; 2nd—(good) 124−103; 3rd (fair)—102−87; 4th (poor)—86−75 and 5th (reject) < 75.

The formula based on NDF for the calculation of DMI (3) potential as a percent of body weight is as follows:

DMI (%) = 120/(NDF%)(3)

The dry matter digestibility used in this equation was determined according to the method of Tilley and Terry [13], modified by Alexander and McGowan [14].

### 2.6. Statistical Analysis

Assumptions of normality and homogeneity of variance were checked with Shapiro–Wilk and Levene’s tests, respectively. The data were analyzed by one-way analysis of variance and means. The means were compared for significance using Duncan’s Multiple Range Test. When normal distribution or homogeneity of variances was not verified, the statistical differences were determined using the non-parametric Kruskal–Wallis’s test being adjusted using the Bonferroni correction. All statistical analyses were performed using the IBM SPSS Statistics 27 program (SPSS Inc., Chicago, IL, USA). Values were expressed as mean ± standard mean error (SEM) and comparisons were considered statistically significant if the *p*-value was lower than 0.05.

## 3. Results

### 3.1. Chemical Composition, Nutritive Value, and Energy Contents

The results of the chemical composition and dry and organic matter digestibility are presented in Table 1. It was observed that *A. melanoxylon*, *A. donax*, and *H. gardnerianu* with 168.6 ± 3.6, 168.6 ± 1.2, and 120.3 g‧Kg^−1^ DM, respectively, present higher values of CP when compared to the remaining plants. The minimum value of CP registered was 65.4 ± 2.1 g‧Kg^−1^ DM for *C. japonica*. *H. gardnerianum* (715.5 ± 3.4 g‧Kg^−1^ DM) and *A. donax* (703.4 ± 1.4 g‧Kg^−1^ DM) had high levels of NDF. On the other hand, *E. globulus* and *P. undulatum* were, of the species studied, those with the lowest NDF values, with 307.9 ± 3.1 and 380.2 ± 8.2 g‧Kg^−1^ DM, respectively.

*A. melanoxylon* was the plant with the highest ADF (522.1 ± 4.9 g‧Kg^−1^ DM) and ADL (464.9 ± 7.7 g‧Kg^−1^ DM) content. The minimum value registered for the ADF was 288.7 ± 3.2 g ‧Kg^−1^ DM for the *E. globulus*, while the lowest value registered for ADL was for the *A. donax* with 31.6 ± 2.2 g‧Kg^−1^ DM. Regarding the ether extract (EE), *E. globulus* was the plant that had the highest content with 80.4 ± 3.2 g‧Kg^−1^ DM.

After determining the in vitro dry matter digestibility *o* (DMD) contents of the non-conventional forages used in the experiment, it was found that they varied between 23.11 ± 1.74% and 60.44 ± 1.22% (Table 1). Regarding the values observed for the in vitro organic matter digestibility in vitro (OMD), the maximum value was obtained for the *A. donax* at 59.62 ± 1.41% while the *E. globulus* presented the minimum percentage of OMD at 21.16%. We must highlight the *A. donax* which was the only plant that presented a digestibility value ‘above 50%, in the DMD and the OMD, while in the other plants the digestibility values were very low, never exceeding 40%.

The RFV of the unconventional forages in the study is presented in Table 2. The RFV was lowest in *H. gardnerianum* and highest in *P. undulatum*. Based on the standards assigned by the Hay Market Task Force of the American Forage and Grassland Council, most of the forage understudy had a very low-quality roughage class, with an RFV below 75%. Only two plants, *P. undulatum*, and *A. donax* had a grade 3 and 4, respectively.

The minimum GE value was 15.68 ± 0.09 MJ‧Kg^−1^ for the *H. gardnerianum* and the maximum 19.93 ± 0.09 MJ‧Kg^−1^ by *E. globulus*, which observed significative differences (*p* < 0.05) to the other plants.

Average values of gross, digestible, and metabolizable energy of each species.

### 3.2. In Vitro Gas Production

The volume of gas produced in vitro from the different plants, during the 96 h of incubation, varied between 1 mL at 4 h for all plants and 34 mL 200 mg^−1^ DM for *A. donax* at 96 h, this being the plant that produced, on average, the highest volume accumulated over time (95.7 mL 200 mg^−1^). *A. melanoxylon* was the plant that produced the lowest volume of gas accumulated during the test with a volume of 21 mL 200 mg^−1^ DM accumulated gas.

**Figure 1:**
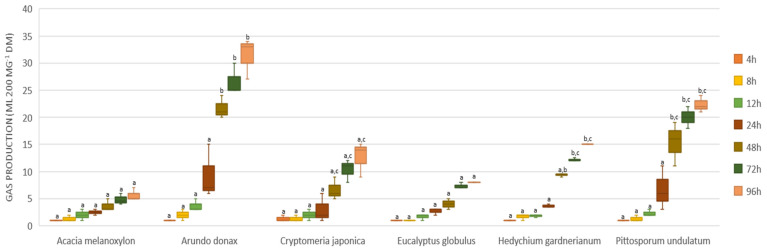
In vitro gas production during incubation of unconventional feeds in buffered rumen fluid at 96 h. Box-plot presentation showing the median, the middle 50% of the data (box), and gas volume ranges. Error bars represent maximum and minimum values per sample, while different letters indicate significant differences among the species, with the *p*-value significant at *p* ≤ 0.05.

As we can observe in Figure 1, there were no significant differences between the plants in the first 24 h of incubation. After 48 h of fermentation, we observed an increase in gas production for *A. donax* compared and the other plants, with significant differences between *A. donax* and *A. melanoxylon*, *C. japonica*, and *E. globulus* during the period between 48 and 96 h. At 72 and 96 h, *P. undulatum* and *H. gardnerianum* registered significant differences between *A. melanoxylon* and *E. globulus*.

The fitted values of in vitro gas production for these unconventional forages (Table 3) show that the initial time of fermentation (Lag Time) varies significantly from plant to plant, ranging between 0 h to 4.8 h.

The kinetics of gas production and the estimated parameters are given in Table 3. Gas production at 96 h was lower in *A. melanoxylon* and higher in *A. donax* with 5.85 mL and 31.56 mL 200 mg^−1^ DM, respectively. The intercept value (a) from the soluble fractions was lowest in *A. donax* and highest in *E. globulus*. In asymptote (b) which represents the fermentation of the insoluble fractions, the minimum value obtained in *A. melanoxylon* and highest in *A. donax*. The forage with the highest gas production potential (a + b) was *A. donax* with 42.39 mL, while the lowest was 7.28 mL observed for *A. melanoxylon*, a statistically significant (*p* < 0.05) difference. *E. globulus* (0.003 mL‧h^−1^) was the plant that presented the lowest fermentation rate (c).

## 4. Discussion

The collected samples were sufficiently representative of the individual species in the sampling area to allow for the investigation of variability between species in terms of chemical composition and food value.

### 4.1. Chemical Composition

The chemical composition of unconventional forages is influenced by several factors, including genotype, stage of maturity, and harvest time [22]. DM represents the share of plant cloth that remains after drying and is composed of carbohydrates (fibrous and non-fibrous), proteins, fats, minerals, and pigments, among others [23]. In all the evaluated plants, DM content varies between 134.0 and 484.4 g‧Kg^−1^ DM.

*H. gardnerianum* showed considerably lower DM content compared to other species, which can be explained by this being the only herbaceous species involved in the study. Among the woody plants, DM varied between 334.3 and 488.4 g‧Kg^−1^ DM, values below those reported by other authors [24,25]. These values may be related to the period of sample collection, since this test was carried out during the winter, when there is greater availability of water in the leaves, decreasing the DM levels [26]. The CP results showed that *C. japonica* and *P. undulatum* with 65.4 ± 2.1 g‧Kg ^−1^ DM and 79.6 ± 2.0 g‧Kg ^−1^ DM values of CP, respectively. These levels are insufficient for ruminal microorganisms to degrade fiber at maximum capacity, as CP levels greater than 80 g‧Kg^−1^ DM are required [27]. However, Van Soest [28] states that the minimum level of CP necessary for normal rumen functioning is 70 g‧Kg^−1^ DM. If we consider this the reference value, only *C. japonica* is below this value. Only two plants, *A. melanoxylon* (168.6 ± 1.2 g‧Kg^−1^ DM) and *A. donax* (168.6 ± 3.6 g‧Kg^−1^ DM) presented CP values above the 150 g‧Kg^−1^ DM required for optimal growth and lactation for dairy cattle [29]. The CP values obtained for *H. gardnerianum* (120.3 ± 4.7 g‧Kg^−1^ DM) were higher than those found by [30] who recorded protein levels of 77.53 g‧Kg^−1^ DM but close to those reported by [31], which were 130.0 g‧Kg^−1^ DM. Protein is one of the main limiting nutrients in livestock. Alternative foods, for the most part, are used as roughage in the base diet, but as a rule, they have low quality with low crude protein content. This situation reduces nitrogen retention in animals and increases their demand, especially in tropical climates [32].

Another important component of the ruminant diet is fiber. Fiber represents the fraction that is partially digestible in the gastrointestinal tract of herbivorous animals and is made up of complex polysaccharides, such as cellulose, hemicellulose, and pectin, as well as lignin, which is rich in phenolic compounds [33]. Through the determination of NDF, ADF, and ADL, we can obtain information about the different fractions of the cell wall (cellulose, hemicellulose, lignin, silica, and insoluble nitrogen compounds). Cellulose and hemicellulose contents are slowly fermented by the rumen microbiota and contribute as a source of metabolic energy [34]. The NDF is composed of cellulose and hemicellulose and lignin, which is an important parameter to stabilize the rumen pH, through the production of saliva. When the NDF content of the diet is greater than 55%, there is evidence of a reduction in food intake [35]. The results showed a great variability of NDF among the plants., However, three of these species, *C. japonica*, *E. globulus*, and *P. undulatum*, have NDF contents lower than 55%. This does not compromise the use of these plants in grazing cattle, as they do not affect the digestibility of the diet. The value obtained for ADF in *P. undulatum* was similar to that found by [36], while for *A. melanoxylon* the determined ADF content obtained (522.1 g‧Kg^−1^ DM) is higher than that presented by Dias et al. [37], which was 311.8 g‧Kg^−1^ DM. ADF is composed of cellulose, lignin, and proteins that limit the degradation of carbohydrates in the cell wall at the rumen level [34], and this value is used to estimate diet digestibility, energy availability, and consumption potential of forage species. Thus, the lower the ADF content of the forage, the higher its nutritional quality and energy levels [38].

The lignin content of 464.9 ± 3.8 g‧Kg^−1^ DM present in *A. melanoxylon* stands out among the various unconventional forages. The high value of ADL will correspond to a low percentage of in vitro DMD (27.02 ± 1.26%), because lignin is difficult to digest, with a negative correlation between lignin concentration and digestibility [39].

The fraction of ash present in plant tissues corresponds to the inorganic mineral component (non-incinerated) that plants absorb the majority from the soil. *H. gardnerianum* was the species that presented the highest ash content 113.3 g‧Kg^−1^ DM, and this herbaceous plant has greater contact with the soil, with a greater probability of contamination since some of the leaves may be in direct contact with the ground. For a better understanding of the mineral part, it was necessary to determine the individual profile of the main minerals present in the ash fraction.

In vitro DMD is an important metric as it represents the proportion of plant material that can be digested by ruminants and is considered one of the main criteria for evaluating the usefulness of food used in animal nutrition [40]. Except for *A. donax*, which presented 60.44% in vitro DMD, all the other plants presented in vitro DMD contents below 50%, the minimum digestibility value for the animals’ maintenance needs [41]. However, authors such as Nastis et al. [42] report that there is an underestimation of the digestibility of shrubs and trees, which may be related to the presence of secondary metabolites, such as saponins or tannins, which negatively influence the in vitro values of digestibility. Tannins have also been associated with low levels of palatability, but low and medium levels can be tolerated by ruminants [43]. The variation in in vitro DMD values is related to the chemical composition of the plant and is generally greater in plants with high CP content and lower fiber values [44]. The presented gross energy levels result in *C. japonica* with 19.5 ± 0.10 MJ‧Kg^−1^ and *E. globulus* (19.93 ± 0.09 MJ‧Kg^−1^), obtaining the highest levels of gross energy. We can associate the GE levels these plants with the high content of essential oils present in *E. globulus* and *C. japonica*.

### 4.2. Relative Feed Values

In this study, most of the studied unconventional forages studied were considered of no interest to animal feed, according to the Quality Grading Standard assigned by The Hay Marketing Task Force of the American Forage and Grassland Council. However, we verified that *P. undulatum* presents a significant difference compared to the other plants in relative values of feed (*p* < 0.05) and fits into a class of fair-quality roughage. Additionally, according to the same scale *A. donax*, with 79.94%, is classified as a poor-quality feed.

### 4.3. Gas Production

The accumulated volume of gas production (Figure 1), the fitted values, and the parameters of the gas production kinetics model (Table 3) showed a variation between different samples, indicating differences in the rate of fermentation characteristics of the feeds.

As expected, the foods with the highest %DMD, *A. donax* and *P. undulatum*, were also the ones that showed the highest accumulated gas production at 96 h, with 31.53 and 22.65 mL 200 mg^−1^ DM, respectively. The observed low in vitro gas production in *A. melanoxylon* and *E. globulus* (Figure 1) might be explained by the presence of high concentrations of total phenols or condensed tannins, as were referred to these plants by Lee et al. [44], and could interact with the protein complexes, being a major cause of the resistance of this species to bacterial decomposition. The biggest significant difference observed in total in vitro gas production was between *A. donax* and *A. melanoxylon*, *E. globulus*, and *C. japonica* (*p* < 0.05). Although *A. donax* has a high rate of ruminal degradation, several authors argue that the palatability of *A. donax* is low, resulting in a low voluntary intake even when ruminants ingest young plant leaves [45].

The negative “a” value observed in forages such as *A. donax*, *P. undulatum*, or *C. japonica*, does not adapt to the concept of gas volume from the soluble and immediately fermentable fraction, but it is due to a deviation from the exponential function in the initial phase of gas production to the fitted data [46].

*P. undulatum’s* gas production from the insoluble fraction “b” observed in this trial (33.33 mL) was identical observed by Dias et al. [37]. l. In a study carried out on *A. donax* obtained a higher “b” was obtained (49.65 mL) [32] than that observed in this work (45.21 mL). *H. gardnerianum* showed, in the same parameter, a value of 37.69 mL higher than that obtained by Moselhy et al. [30] which was 35.30 mL, but significantly lower than the 57.04 mL indicated by Dias et al. [37] for this species. This variation can be explained by the season of the year in which the samples were harvested, the state of maturity of the plant, or the place where samples were collected.

The potential extent of gas production (a + b) was significantly different between *C. japonica* and *A. melanoxylon* compared to other plants (*p* < 0.05). These plants showed lower gas production. *A. donax* was the plant that showed the highest gas production potential, contrary to what was reported by Cone et al. [47] that refer that roughages with a high CP content normally produce less gas during fermentation, even if their gas production extent (a+b) is high because the fermentation of the protein produces ammonia, which influences the balance of the carbonate buffer by neutralizing the ions H^+^ of volatile fatty acids without the release of CO_2_. In the fermentation parameter (a + b) we recorded a volume of 37.74 mL for *H. gardnerianum* and 30.79 mL for *P. undulatum*, which differ from the results obtained by Moselhy et al. [30] who obtained a potential for the gas production of these plants of 25.32 and 37.28 mL, respectively. These authors say that these two plants have an inhibitory effect on in vitro gas production.

This study focused only on the nutritional composition of plants and their digestibility; however, further research is needed to identify the presence of secondary metabolites before it is fully recommended. These plants must also undergo animal response testing to assess their acceptability and the effect on animal performance and the quality of their products.

## 5. Conclusions

After analysis of the different parameters under evaluation such as chemical composition, digestibility, and potential gas production, it was concluded that all the species under study were nutritionally poor. However, among the six species studied two species stand out, *A. donax* and *P. undulatum*. *P. undulatum*, despite having a low protein content, has a fair rate of quality RFV forage, which allows it to be an alternative source of fiber for ruminants. Overall, *A. donax* and *P. undulatum* showed the best results and can be supplied to ruminants as roughage in periods of food scarcity. Nevertheless, before testing on animals in vivo, further studies will be needed to identify possible antagonist substances, and physical and/or chemical treatments to the unconventional forages under study are also needed to increase the exposure of fibrous components to ruminal degradability.

## Figures and Tables

**Table 1:** Chemical Composition of the unconventional forages.

Forages	Dry Matter (g‧Kg^−1^)	g Kg^−1^ DM						% DMD	% OMD	GE (MJ‧kg^−1^ DM)
		CP	NDF	ADF	ADL	EE	Ash			
*A. melanoxylon*	395.1 (±9.3)	168.6 (±1.2)	640.8 (±4.3)	522.1 (±4.9)	464.9 (±7.7)	20.2 (±2.4)	52.2 (±0.0)	27.02 (±1.26)	24.58 (±1.18)	16.07 (±1.62)
*A. donax*	244.0 (±6.1)	168.6 (±3.6)	703.4 (±1.4)	334.4 (±6.4)	31.6 (±2.2)	20.3 (±1.3)	107.3 (±1.2)	60.44 (±1.22)	59.62 (±1.41)	16.96 (±1.18)
*C. japonica*	364.5 (±4.3)	65.4 (±2.1)	467.7 (±1.0)	334.3 (±5.2)	184.5 (±4.0)	63.6 (±5.9)	46.7 (±7.2)	30.81 (±0.49)	28.78 (±0.84)	19.5 (±1.90)
*E. globulus*	484.4 (±5.6)	81.0 (±2.2)	307.9 (±3.1)	288.7 (±3.2)	192.4 (±5.9)	80.4 (±3.2)	45.1 (±1.4)	23.11 (±1.74)	21.16 (±1.57)	19.93 (±2.09)
*H. gardnerianum*	134.0 (±3.6)	120.3 (±4.7)	715.5 (±3.4)	360.9 (±1.6)	91.3 (±9.7)	22.5 (±0.9)	113.3 (±2.2)	36.39 (±1.70)	29.19 (±1.49)	15.68 (±1.58)
*P. undulatum*	334.3 (±6.1)	79.6 (±2.0)	380.2 (±8.2)	355.9 (±6.7)	193.2 (±7.6)	30.2 (±4.1)	78.7 (±1.1)	37.65 (±0.91)	34.00 (±1.45)	18.63 (±1.28)

Results presented as a mean (± standard deviation.) CP—Crude Protein; NDF—Neutral Detergent Fiber; ADF—Acid Detergent Fiber; ADL—Acid Detergent Lignin; EE—Ether Extract; DMD—Dry Matter Digestibility; OMD—Organic Matter Digestibility; GE – Gross Energy. *p*-value significant at *p* ≤ 0.05.

**Table 2:** Relative feed values and qualities of the unconventional forages.

Forages	DMD (%)	DMI (%BW)	RFV (%)	Classification
*Acacia melanoxylon*	27.02 ^a^	1.87 ^a^	39.22 ^a^	5
*Arundo donax*	60.44 ^b^	1.71 ^a^	79.94 ^b^	4
*Cryptomeria japonica*	30.81 ^a^	2.57 ^ab^	61.29 ^ab^	5
*Eucalyptus globulus*	23.11 ^a^	3.90 ^b^	69.82 ^ab^	5
*Hedychium gardnerianum*	36.39 ^a^	1.68 ^a^	34.31 ^a^	5
*Pittosporum undulatum*	37.65 ^a^	3.16 ^b^	92.12 ^c^	3
SEM	2.62	0.33	7.23	
*p*-value	≤0.05	≤0.05	≤0.05	

Means with different letters in the same column are significantly different (*p* < 0.05); SEM—standard error of means. *p*-value significant at *p* ≤ 0.05. According to the Quality Grading Standard assigned by The Hay Marketing Task Force of the American Forage and Grassland Council, the RFV was assessed as roughages based on: prime >151; 1st (premium)—151−125; 2nd(good)—124−103; 3rd (fair)—102−87; 4th (poor)—86−75 and 5th (reject) < 75.

**Table 3:** Fitted values and parameters of kinetics in vitro gas production gas after 96 h incubation of the unconventional forages.

Forages	Fitted Values of Gas Production (mL 200 mg^−1^ DM)							Parameters of Gas Production					
	4 h	8 h	12 h	24 h	48 h	72 h	96 h	a	b	a + b	c	Lag Time	RSD
*A. melanoxylon*	0.98 ^A^	1.37 ^A^	1.74 ^A^	2.71 ^A,D^	4.18 ^A^	5.17 ^A^	5.85 ^A^	0.56 ^A^	6.72 ^A^	7.28 ^A^	0.016 ^A^	0	0.31
*A. donax*	0.21 ^B^	2.25 ^B^	4.57 ^B,C^	10.74 ^B^	20.23 ^B^	26.88 ^B^	31.53 ^B^	−2.82 ^B^	45.21 ^c^	42.39 ^B^	0.015 ^A^	4.3	1.44
*P. undulatum*	0.34 ^B^	1.42 ^A^	3.09 ^B,C^	7.53 ^C^	14.4 ^C^	19.24 ^B^	22.65 ^C^	−2.21 ^B^	33.00 ^B^	30.79 ^B^	0.014 ^A^	4.8	1.1
*E. globulus*	0.99 ^A^	1.34 ^A^	1.69 ^A^	2.71 ^A,D^	4.65 ^A^	6.46 ^A^	8.16 ^A^	0.63 ^A^	31.88 ^B^	32.51^B^	0.003 ^B^	0	0.51
*C. japonica*	0.20 ^B^	1.13 ^A^	1.97 ^A^	4.01 ^A,D^	6.58 ^A^	7.94 ^A,C^	8.67 ^A^	−0.83 ^D^	10.32 ^A^	9.49 ^A^	0.264 ^C^	3.2	1.58
*H. gardnerianum*	0.84 ^A^	1.62 ^A^	2.37 ^A,B,C^	4.55 ^A,D^	8.52 ^A,C^	12.01 ^C^	15.09 ^D^	0.05 ^C^	37.69 ^B,C^	37.74 ^B^	0.005 ^B^	0	0.46
SEM	0.09	0.19	0.24	0.22	0.29	0.14	0.11	0.02	0.12	0.09	0.007		
*p*-value	≤0.05	≤0.05	≤0.05	≤0.05	≤0.05	≤0.05	≤0.05	≤0.05	≤0.05	≤0.05	≤0.05		

Means within a column with different capital letters differ significantly, *p*-value significant at *p* ≤ 0.05.a: gas production from the immediately soluble fraction (mL); b: gas production from the insoluble fraction (mL); a + b: potential gas production (mL); c: the gas production rate constant for the insoluble fraction (mL/h); lag time expressed in hours.

## Data Availability

The data presented in this study are available on request from the corresponding author.
